# Exercise interventions of ≥8 weeks improve body composition, physical function, metabolism, and inflammation in older adults with stage *I* sarcopenic obesity: a systematic review and meta-analysis

**DOI:** 10.3389/fnut.2025.1575580

**Published:** 2025-08-12

**Authors:** Huiting Wei, Jiabao Zhang, Kaiyin Cui, Hao Su

**Affiliations:** ^1^Sport Science School, Beijing Sport University, Beijing, China; ^2^Beijing Higher School Engineering Research Center of Sport Nutrition, Beijing, China

**Keywords:** exercise, stage *I* sarcopenic obesity, body composition, physical function, metabolism, inflammation

## Abstract

**Introduction:**

This study aimed to assess the benefits of ≥8-week exercise interventions for stage *I* sarcopenic obesity (SO) without complications.

**Methods:**

Randomized controlled trials (RCTs) published from 2004 to July 2024 were searched in PubMed, Embase, Web of Science, the Cochrane Library, and EBSCO. Publication bias was assessed using funnel plots and Egger’s test. The search strategy was prospectively registered in PROSPERO (ID number: CRD42024619070). Heterogeneity (I^2^ > 50%) was managed using random-effects models.

**Results:**

Fifteen parallel-group RCTs involving 623 elderly adults (aged ≥60 years) were included. Exercise significantly reduced BMI (MD = −1.35, *p* < 0.0001), with combined exercise (CE) being the most effective (MD = −1.25, *p* < 0.001). Body fat percentage decreased (MD = −0.52, *p* < 0.00001) with CE outperforming resistance training (RT). No significant changes in fat mass or muscle mass were found (fat mass, *p* = 0.19; appendicular skeletal muscle mass, *p* = 0.88; and appendicular skeletal muscle mass index, *p* = 0.86). Physical function (grip strength, gait speed, and the timed Up and Go test) improved significantly (*p* < 0.00001); RT and CE enhanced muscle strength, with RT being superior (MD = 3.43 vs. 2.64 for CE, both *p* < 0.00001). Additionally, CE lowered insulin (MD = −1.73, *p* < 0.05) and total cholesterol (MD = −0.38, *p* < 0.05) levels, with marginal interleukin-6 reduction (MD = −0.51, *p* = 0.08). Other metabolic and inflammatory markers (glucose, triglycerides, high-density lipoprotein cholesterol, low-density lipoprotein cholesterol, tumor necrosis factor-*α*, and C-reactive protein) remained unchanged.

**Discussion:**

≥8-week exercise improves body composition in stage *I* SO, with CE being the most effective for fat loss. Physical function improves with both RT and CE, and RT is better for muscle strength, while CE benefits metabolism and inflammation. We recommend that CE (≥3 times/week, 45 min/session) be used for high inflammation and RT (2–3 times/week, 60–80% of 1-RM) for low inflammation. Based on observed data trends, promoting a CE model of three aerobic exercises + two RT sessions weekly is advisable, with the intensity adjusted to 40–50% 1-RM for stage *I* elderly patients. Future research needs large-sample, long-term RCTs with subgroup analyses and exercise-nutrition combinations.

**Systematic review registration:**

The search strategy was prospectively registered in PROSPERO (ID number: CRD42024619070).

## 1 Introduction

With the acceleration of the global aging process, the proportion of the elderly population within the total population is increasing every year, and the resulting metabolic health problems have become the focus of social attention (1). Of these, sarcopenia and obesity are two global concerns associated with poor health outcomes in older adults (2). Sarcopenic obesity (SO), a complex syndrome commonly seen in the elderly, is characterized by the coexistence of decreased muscle mass and increased fat mass, which poses a greater threat than simple obesity and sarcopenia (3). One study further noted that sarcopenia and SO are common phenotypes in older adults and are associated with all-cause mortality (4). Research groups in Europe and Asia have reached a consensus regarding the definition and diagnostic criteria for sarcopenia. The global prevalence of sarcopenia is estimated to be approximately 10% in adults aged ≥60 years (5), with Asian populations ranging from 5.5 to 25.7% among individuals aged ≥40 years (6). Due to the complexity of the etiology of SO and the difficulty in confirming the diagnosis, no official global prevalence has been reported. However, a systematic evaluation and meta-analysis yielded a global prevalence of 11% for SO in adults aged ≥ 60 years (7). However, its prevalence may be low in some countries. For example, the German KORA-Age study (8) reported a 4.5% SO prevalence, which is lower than 6.0% in the Chinese population (9), possibly due to dietary and lifestyle differences.

SO is closely associated with insulin resistance, abnormal lipidemia, lack of exercise, inflammation, hypertension, low spontaneous physical activity, inadequate protein and energy intake, low levels of exercise training, and aging, which are the most significant risk factors (10). The occurrence of SO not only leads to metabolic disorders, thereby increasing the prevalence of various chronic diseases, such as hypertension, diabetes, high cholesterol, and cardiovascular diseases, but also results in a decline in motor ability, an increased risk of osteoporosis and arthritis, and an elevated risk of falls and injuries, which severely affects the quality of life of the elderly (11–13). Hence, despite not being highly prevalent, the severity of SO should not be underestimated. However, the current assessment standards for SO are inconsistent. According to the ESPEN and EASO Consensus Statement in 2022 (14), experts divided the stages of SO into two phases. In stage *I*, the altered body composition and skeletal muscle functional parameters of SO did not trigger any complications. In stage *II*, there is at least one complication resulting from altered body composition and skeletal muscle functional parameters, such as metabolic diseases, disabilities caused by high-fat mass and/or low-muscle mass, and cardiovascular and respiratory diseases.

The current assessment standard for SO is a combination of the assessment criteria for obesity and sarcopenia. Obesity is assessed internationally using the body mass index (BMI) (15) or body fat percentage (BF%) (16). The European Working Group on Sarcopenia in Older People 2 (EWGSOP2) (17) defined sarcopenia as an age-related loss of muscle mass, low muscle strength, or low physical performance, and proposed diagnostic cut-offs for components. However, many studies have failed to standardize the criteria for research subjects. It is necessary to precisely define the assessment criteria for SO to provide a theoretical foundation for subsequent in-depth research.

Insulin resistance is the central mechanism underlying SO and is associated with diverse cardiometabolic disorders (18). Patients with SO often exhibit detrimental changes in their lipid profiles, such as increased triglycerides (TG), decreased high-density lipoprotein cholesterol (HDL-C), and potentially elevated or oxidatively modified low-density lipoprotein cholesterol (LDL-C). High levels of age-related inflammatory markers are detected in most elderly individuals even in the absence of risk factors and clinically active diseases (19). C-reactive protein (CRP) and interleukin-6 (IL-6) are key inflammatory markers associated with the aging process (20). Enlarged adipocytes secrete large quantities of inflammatory factors such as tumor necrosis factor-*α* (TNF-*α*) and IL-6 (21). These inflammatory factors not only induce an inflammatory response in adipose tissue but also affect systemic tissues, including the liver and muscles, and negatively impact glucose and lipid metabolism. Therefore, surveillance of inflammatory cytokines is crucial, as it can provide a theoretical basis for further research on the mechanisms linking metabolism, inflammation, and SO.

Traditional approaches, such as nutritional therapy and surgical treatment, have relatively weak therapeutic effects and high costs for treating SO. Drugs that can counteract muscle loss during aging or disease are limited, and various new treatment methods are still in the exploratory stage (22). Exercise, as a non-pharmacological, cost-effective intervention, offers health benefits such as improved body composition and increased strength, and has great potential to improve the health of older adults with SO. Although different exercise interventions (either alone or in combination) and different exercise intensities and durations produce different results, previous studies have revealed some major limitations. On the one hand, due to the disease characteristics of SO, comorbidities in patients with stage *II* SO may confound the effects of exercise. On the other hand, the effects of aerobic exercise (AR), resistance training (RT), or combined exercise (CE) are insufficiently quantified due to the diversity of exercises; additionally, the duration of most interventions is poorly defined, and long-term data are lacking.

Previous studies on SO have mostly focused on improvements in specific indicators like body composition or exercise performance, neglecting others. Some also failed to differentiate disease stages in participants, which may hinder interpreting exercise intervention benefits. For example, Laura et al. (23) noted that exercise interventions are effective in enhancing physical function. They further suggested that future research should investigate the effects of different exercise modalities (23); however, their study did not focus on the changes in metabolism and inflammation. Chun-De et al. (24) noted the benefits of elastic resistance exercise on body composition and physical function in women with SO. Some participants had metabolic diseases, such as hypertension, hyperlipidemia, and diabetes, which may affect the interpretation of the exercise intervention’s effects (24). In this meta-analysis, elderly participants were defined as individuals aged ≥60 years in accordance with EWGSOP2 criteria (17), as muscle decline accelerates significantly after the age of 60. Studies with intervention durations shorter than 8 weeks were excluded because of the instability of short-term effects. Overall, this study included ≥8-week exercise interventions in patients with stage *I* SO and systematically evaluated their effects on body composition, physical performance, glucose and lipid metabolism, and inflammatory biomarkers, thereby providing evidence for early therapeutic windows in SO.

## 2 Materials and methods

### 2.1 Research design and registration

This meta-analysis was conducted in accordance with the Preferred Reporting Items for Systematic Reviews and Meta-Analyses (PRISMA) (25). Through systematic literature retrieval, screening, extraction, and analysis of relevant studies, a comprehensive conclusion regarding the effects of exercise was reached. The study protocol was registered in PROSPERO with the ID number CRD42024619070.

### 2.2 Search strategies

A comprehensive and systematic search was conducted to identify eligible studies published from 2004 to July 2024. Databases including PubMed, Embase, Web of Science, and the Cochrane Library were searched using terms related to exercise (e.g., exercise, training, aerobic exercise, endurance training, resistance training, strength training, circuit training, combined training, moderate-intensity continuous training) and sarcopenic obesity (e.g., sarcopenia, sarcopenic obesity, obesity with sarcopenia), along with terms related to study design (e.g., randomized controlled trial, randomized, placebo). The search strategy was prospectively registered with PROSPERO (CRD42024619070). Gray literature was excluded, which may have introduced a publication bias (e.g., underreporting of null results). The literature search strategy for each database is provided in the Appendix.

### 2.3 Study selection

Two trained researchers independently performed the literature screening. The titles and abstracts of all relevant studies were retrieved, and those that were clearly irrelevant were excluded. Subsequently, the full texts of the remaining studies were obtained, a meticulous review was conducted, and the studies were screened according to predetermined inclusion and exclusion criteria. The inclusion criteria were as follows: (i) study participants were adults aged ≥60 years with stage *I* SO; (ii) the intervention was a comparison between an exercise intervention and a non-exercise intervention; (iii) outcome indicators included at least one of body composition, muscle function, metabolic markers, or inflammatory biomarkers; (iv) the study design was an RCT; and (v) the exercise training duration was ≥8 weeks. The exclusion criteria were as follows: (i) duplicate or unpublished studies; (ii) incomplete data, inability to extract valid data, or inability to obtain the full text; (iii) non-English language; and (iv) non-original research (e.g., conference proceedings, guidelines, abstracts, reviews, case reports, and commentaries). The inclusion criteria are listed in Table 1.

**Table 1:** Literature inclusion criteria.

PICOS element	Criteria
Population	≥60 years, stage *I* SO (complication-free), obesity via BMI/BF%, sarcopenia via EWGSOP2/AWGS
Intervention	One or more exercise interventions, ≥ 8 weeks
Comparison	Non-exercise control (usual care/health education)
Outcomes	Primary: body composition (BMI, BF%, fat mass, ASMI), physical performance (grip strength, gait speed, TUG, knee extension); Secondary: metabolic markers (glucose, insulin, TC, TG, HDL-C, LDL-C), inflammatory cytokines (TNF-*α*, CPR, IL-6)
Study design	Randomized controlled trial (RCT)

### 2.4 Data extraction

For the final included studies, two researchers independently extracted relevant data using a pre-designed data extraction form Table 2, including the following basic information: (i) authors, publication year, and study location; (ii) characteristics of the study population (grouping, sample size, age, and sex); (iii) definition of SO and tools for assessing body composition; (iv) type and duration of the exercise intervention; and (v) outcomes. Two independent reviewers assessed the bias and disagreements were resolved by a third expert through consensus meetings. Assessments were conducted on 159 papers: Assessor A included 20 and excluded 139, Assessor B included 22 and excluded 137, and 18 were included by both A and B. The Cohen’s kappa value was approximately 0.836, indicating a high degree of consistency.

**Table 2:** Characteristics of studies.

Author and year	Country	Study design	Groups (sample size)	Age (sex)	Definition of sarcopenic obesity		Assessment tool of body composition	Type of exercise intervention	Time point of measurement	Outcomes
					Sarcopenia	Obesity				
Balachandran 2014 (26)	America	Parallel-group RCT	Hypertrophy (9)	71 ± 8.2 (F)	SMI < 10.76 kg/m^2^ in men and 6.76 kg/m^2^ in women or gait speed <1 m/s or GS < 30 kg for men and 20 kg for women	BMI > 30 kg/m^2^	BIA	RT	Baseline: 0 week Posttest: 15th week	SPPB, leg press power, 1-RM of leg press and chest press, BF%, LBM, SMI; PFP-10, GS
			High-speed circuit (8)	71.6 ± 7.8 (Both)						
Kim 2016 (28)	Japan	Parallel-group RCT	Exercise (35)	81.4 ± 4.31 (F)	SMI < 5.67 kg/m^2^ or GS < 17 kg or WS < l m/s	BF% > 32	BIA	Combined exercise	Baseline: 0 week Posttest: 12th week	BF%, TFM, ASM, GS, KES, WS; TC, TG; IL-6, hs-CRP
			Health Education (34)	81.1 ± 5.11 (F)				Non-exercise		
Maltais 2016 (27)	America	Parallel-group RCT	RT + Nondairy Shake (8)	64 ± 4.9 (M)	SMI < 10.75 kg/m^2^	BMI > 30 kg/m^2^	DXA	RT	Baseline: 0 week Posttest: 16th week	BMI, FM; TG, HDL, LDL, TC, GLU, insulin; IL-6, TNF-*α*, CRP
			RT + Dairy Shake (8)	68 ± 5.1 (M)						
			RT (10)	64 ± 4.5 (M)						
Vasconcelos 2016 (29)	Brazil	Parallel-group RCT	Exercise (14)	72 ± 4.61 (F)	handgrip strength ≤21 kg	BMI ≥ 30 kg/m^2^	NA	RT	Baseline: 0 week Posttest: 10th week	strength, power, SPPB, gait velocity, SF-36
			Control (14)	72 ± 3.61 (F)				Non-exercise		
Chen 2017 (31)	China	Parallel-group RCT	RT (15)	68.9 ± 4.4 (Both)	SMI < 32.5%in men, <25.7% in women	BMI ≥ 25 kg/m^2^ and VFA ≥ 100 cm^2^	BIA	RT	Baseline: 0 week Posttest: 8th week	BMI, PBF%, SMM, ASM/Weight; Grip, BES; IGF-1
			AR (15)	69.3 ± 3.0 (Both)				AR		
			Combination training (15)	68.5 ± 2.7 (Both)				Combined exercise		
			Control (15)	68.6 ± 3.1 (Both)				Non-exercise		
Huang 2017 (32)	China	Parallel-group RCT	ERT (18)	68.89 ± 4.91 (F)	SMI < 27.6%	BF% > 30	DXA	RT	Baseline: 0 week Posttest: 12th week	BMI, SMI, BF%, TFM; T-score, Z-score; TG, HDL, LDL, TC; CRP
			Control (17)	69.53 ± 5.09 (F)				Non-exercise		
Liao 2017 (24)	China	Parallel-group RCT	Experimental (25)	66.39 ± 4.491 (F)	SMI < 7.15 kg/m^2^	BF% > 30	DXA	RT	Baseline: 0 week Posttest: 12th week	FFM, LLM, TFM, BF%; GS, TUG, handgrip
			Control (21)	68.42 ± 5.86 1 (F)				Non-exercise		
Park 2017 (33)	Korea	Parallel-group RCT	Combined exercise (25)	73.5 ± 7.11 (F)	ASM/ weight <25.1%	BMI ≥ 25.0 kg/m^2^	DXA	Combined exercise	Baseline: 0 week Posttest: 24th week	BF%, ASM; GS, sit and reach, MWS; TG, HDL, LDL, TC; hs-CRP
			Control (25)	74.7 ± 5.11 (F)				Non-exercise		
Chiu 2018 (39)	China	Parallel-group RCT	Exercise (33)	79.64 ± 7.36 (Both)	SMI (TSM/BW) < 37.15% in men; <32.26% in women	BF% > 29 in men; BF% > 40 in women	BIA	RT	Baseline: 0 week Posttest: 12th week	skeletal muscle%, ASMI, BF%; GS, total pinch strength, total FIM score
			Comparison (31)	80.15 ± 8.26 (Both)				Non-exercise		
Liao 2018 (37)	China	Parallel-group RCT	Experimental (33)	66.67 ± 4.541 (F)	SMI < 27.6%	BF% > 30	BIA	RT	Baseline: 0 week Posttest: 12th week	BF%, TSM, ALM, LMI, AMI, SMI %; GS, TUG, UE, LE, SF-36
			Control (23)	68.32 ± 6.051 (F)				Non-exercise		
Nabuco 2019 (30)	Brazil	Parallel-group RCT	RT + whey protein (13)	68.0 ± 4.21 (F)	ASM < 15.02 kg	BF% > 35	DXA	RT	Baseline: 0 week Posttest: 12th week	ALST, WC, TFM; 10 MW, KES, chest press; TG, HDL, LDL, TC, GLU, insulin, HOMA-IR; IL-6, TNF-*α*, CRP
			RT (13)	70.1 ± 3.91 (F)						
Liao 2020 (38)	China	Parallel-group RCT	Experimental (20)	72.22 ± 7.751 (F)	gait speed <0.8 m/s	BMI ≥ 24 kg/m^2^	DXA	RT	Baseline: 0 week Posttest: 12th week	arm lean mass, leg lean mass, ASMI, gait speed, WOMAC−PF, UE, LE
			Control (20)	69.79 ± 6.721 (F)				Non-exercise		
Jung 2022 (34)	Korea	Parallel-group RCT	Exercise (14)	75.36 ± 4.501 (F)	ASM/height ≤5.4 kg/m^2^	BF% > 32	DXA	Circuit exercise	Baseline: 0 week Posttest: 12th week	BMI, FFM, ASMI, WHR; TG, HDL, LDL, TC, HOMA-IR
			Control (14)	74.64 ± 5.771 (F)				Non-exercise		
Magtouf 2023 (36)	Tunisia	Parallel-group RCT	Exercise (25)	76.1 ± 3.5 (NA)	handgrip <17 N, gait speed <1.0 m/s	BMI ≥ 30 kg/m^2^	NA	Concurrent Exercise	Baseline: 0 week Posttest: 24th week	BMI, BF%, FBM, LBM, WC, HC; hand grip, GS, TUG, Romberg test
			Control (25)	75.9 ± 5.4 (NA)				Non-exercise		
Jung 2024 (35)	Korea	Parallel-group RCT	Exercise (14)	78.14 ± 3.721 (F)	ASM/height ≤5.4 kg/m^2^	BF% > 32	BIA, DXA	Circuit Exercise	Baseline: 0 week Posttest: 12th week	BMI, FFM, BF%, ASMI; hs-CRP, IL-6, TNF-*α*
			Control (14)	78.21 ± 3.721 (F)				Non-exercise		

RCT, randomized controlled trial; ERT, elastic band resistance training; RT, resistance training; F, female; M, male; SMI, skeletal muscle index; BMI, body mass index; BF%, body fat percentage; DXA, dual-energy X-ray absorptiometry; BIA, bioelectrical impedance analysis; ASM, appendicular muscle mass; FM, fat mass; FFM, fat-free mass; TFM, total fat mass; ALST, appendicular lean soft tissue; TSM, total skeletal muscle; WC, waist circumference; 10 MW, 10 m walk test; KES, knee extensor strength; TC, total cholesterol; TG, triglycerides; HDL, high-density lipoprotein; LDL, low-density lipoprotein; GLU, glucose; IL-6, interleukin-6; IL-8, interleukin-8; CRP, C-reactive protein; TNF-*α*, tumor necrosis factor alpha; IGF-1, insulin-like growth factor-1; HOMA-IR, homeostatic model assessment of insulin resistance; ASMI, appendicular skeletal muscle mass index; WHR, waist-to-hip ratio; GS, grip strength; MWS, maximum walking speed; WS, walking speed; SPPB, short physical performance battery; SF-36, 36-item Short Form Health Survey; SM, skeletal muscle mass; ALM, appendicular lean mass; LMI, lean mass index; TUG, timed up and go; UE, upper extremity; LE, lower extremity; WOMAC-PF, physical function subscale of Western Ontario & McMaster Universities Osteoarthritis Index; HC, hip circumference; 1-RM, one-repetition maximum; LBM, lean body mass; PFP-10, physical functional performance test; NA, not available.

### 2.5 Quality assessment

The Cochrane Risk of Bias assessment tool, which is the standard quality assessment instrument for RCTs, was used to evaluate the quality of the included studies. Sources of bias were evaluated on multiple aspects, including the random allocation method, allocation concealment, assessor-blind method, data integrity, selective reporting of results, and other potential sources of bias. Subsequently, the study quality was categorized as high, medium, or low. During the meta-analysis, the potential influence of the study quality on the results was considered.

### 2.6 Statistical analysis

The characteristics of the included studies were summarized, and the baseline data for each study group were presented in the tables. Measured values and relevant statistics for outcome indicators in both the experimental and control groups at the end of follow-up were listed, including means, standard deviations, and sample sizes. Changes from baseline to post-test in both groups were combined to assess the effects. As all data were continuous variables with the same units, the mean differences (MD) and 95% confidence intervals (CI) were used for statistical analysis. If the units differed, the standardized mean differences (SMD) and 95% CI were computed.

Study heterogeneity was evaluated using the Cochrane Q test and I^2^ statistic. An I^2^ value of 0–25% indicated low heterogeneity, 25–50% indicated moderate heterogeneity, 50–75% indicated high heterogeneity, and >75% indicated very high heterogeneity. A Q test *p*-value ≤0.10 also indicated significant heterogeneity. In the absence of heterogeneity (*I*^2^ = 0% or near 0%, and Cochrane Q test *p* > 0.1), a fixed-effect model was used, with the pooled effect size computed as the weighted average of individual study effect sizes. If significant heterogeneity was present, a random-effects model was used to calculate the pooled effect size and its 95% CI, which is shown as a diamond in the forest plot. The clinical and statistical significance of the pooled effect size is explained. If high heterogeneity persisted, a subgroup analysis was conducted.

Publication bias was evaluated by inspecting funnel plots and was statistically assessed using Egger’s test. Sensitivity analyses were conducted by excluding trials with a high risk of bias to test the robustness of the pooled results. Quantitative synthesis of data was performed using Review Manager software version 5.4.1. For outcomes that could not be pooled, a narrative summary of the results was provided along with an explanation of the limitations.

## 3 Results

### 3.1 Study selection

**Figure 1:**
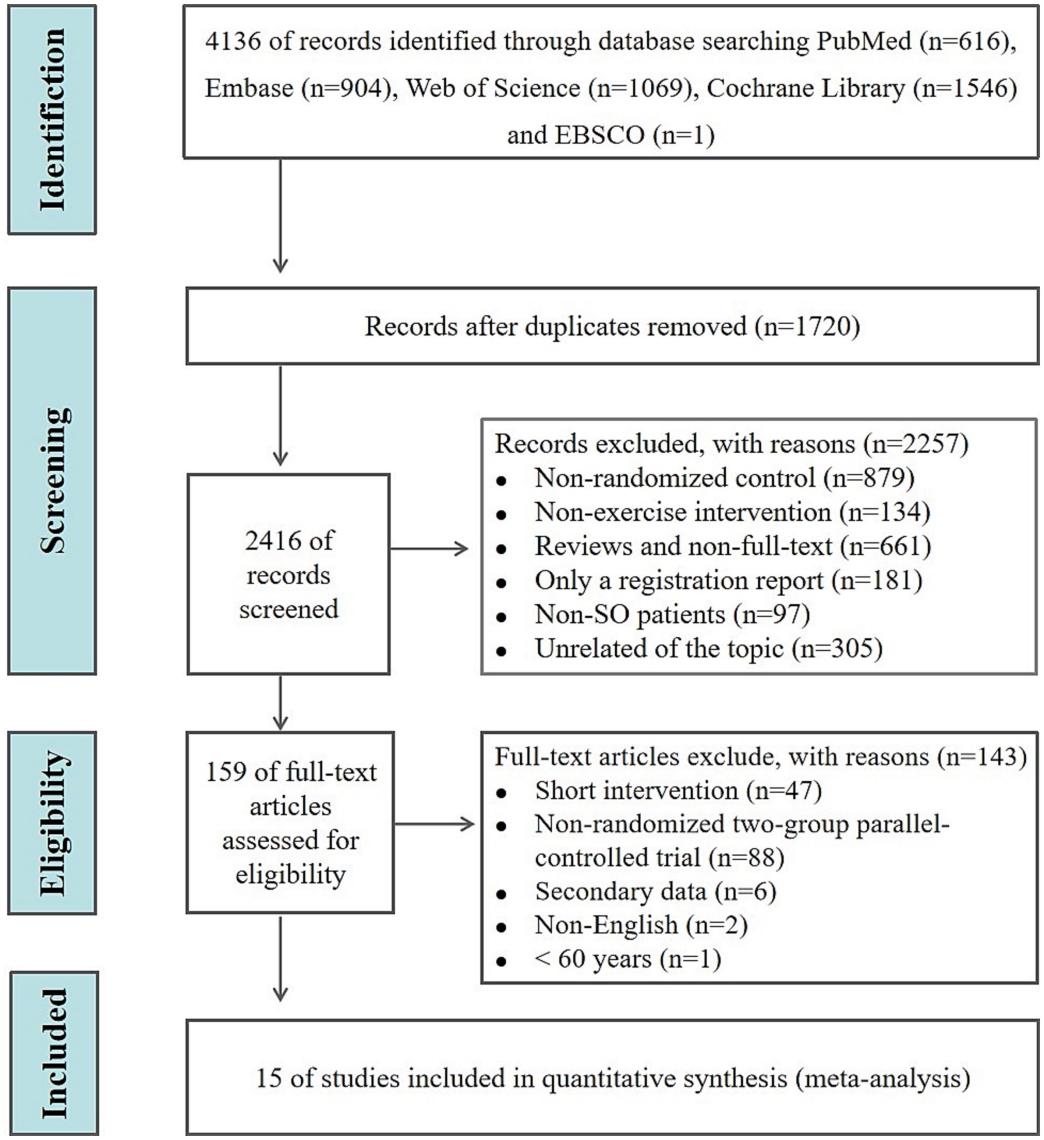
Study selection process.

Based on the search terms, 4,136 original studies were initially identified from the PubMed (*n* = 616), Embase (*n* = 904), Web of Science (*n* = 1,069), the Cochrane Library (*n* = 1,546), and EBSCO (*n* = 1) databases. After removing duplicates, systematic reviews, and meta-analyses, the titles and abstracts of 2,416 studies were screened. A total of 2,257 records were excluded for the following reasons: (i) non-randomized design (*n* = 879); (ii) non-exercise intervention (*n* = 134); (iii) reviews or non-full-text (*n* = 661); (iv) registration reports only (*n* = 181); (v) non-stage *I* SO patients (*n* = 97); (vi) unrelated to the topic (*n* = 305). Following the screening, 159 articles were assessed for eligibility. Among them, 47 studies were excluded due to short intervention duration, 88 were excluded because they were not RCTs, six were excluded because they were conference abstracts or secondary data, two were excluded due to non-English language, and one was excluded because the study participants were younger than 60 years old. Finally, 15 RCTs were included in this meta-analysis (Figure 1).

### 3.2 Study characteristics

Across all included studies, a total of 623 participants were included in the analyses after excluding those lost to follow-up. These studies were published between 2004 and July 2024, with study sites including America (26, 27), Japan (28), Brazil (29, 30), China (24, 31, 32), Korea (33–35), and Tunisia (36). All 15 studies included participants aged ≥60 years. Obesity was defined as BMI (≥24–30 kg/m^2^) or BF% (men >29%, women >30–40%), and sarcopenia was defined by skeletal muscle index (SMI), SMI%, ASM, ASM/height, ASM/weight, grip strength, and gait speed. In terms of gender distribution, 10 studies (24, 28–30, 32–35, 37, 38) recruited only female participants, one study (27) recruited only male participants, three studies (26, 31, 39) recruited both genders, and one study (36) did not specify gender. To assess body composition, five studies (26, 28, 31, 37, 39) used BIA, eight studies (24, 27, 30, 32–35, 38) used DXA, one study (35) used both, and two studies (29, 36) did not specify. Regarding intervention duration, 13 studies (24, 26–30, 32–39), had a duration of 12 weeks or more, while two studies (29, 31) were set to 8–10 weeks. All the studies were designed as two-group, randomized, parallel-controlled trials. Table 2 presents the basic characteristics of the 15 studies.

### 3.3 Exercise protocol

15 RCTs all explored the impact of exercise intervention on stage *I* SO without complications. One study (26) compared hypertrophy training and high-speed circuit training; two studies (28, 33) explored the effects of CE and a control group; eight studies (24, 27, 29, 30, 32, 37–39) compared RT and a control group; one study (31) compared the effects of RT, AR, CE, and control groups; two studies (34, 35) focused on the effects of circuit exercise on SO; and one study (36) focused on the effects of concurrent exercise.

Training frequencies were 2–3 days/week, with intensities ranging from 40 to 85% of the one-repetition maximum (1-RM) or heart rate reserve (HRR). Six studies (26, 29, 31, 36, 38, 39) involved training 2 days/week, while the other 9 studies involved training 3 days/week. Regarding exercise duration per week, six studies (26, 28, 29, 31, 36, 38) had a duration of no more than 120 min, seven studies (24, 27, 32, 33, 35, 37, 39) had a duration of more than 120 min, one study (34) had a duration of 75–225 min, and one study (30) did not elaborate on it. For exercise intensity, five studies (26, 27, 29–31) used one repetition maximum (1-RM), five studies (24, 32, 33, 37, 39) used the rating of perceived exertion (RPE), one study (38) used both 1-RM and RPE, two studies (34, 35) used heart rate reserve (HRR), one study (27) used the maximum heart rate (HRmax), one study (36) used 300 arbitrary units (a. u.), and one study (28) did not specify. One study (27) used high-intensity exercise, seven studies (26, 29, 30, 33–35, 38) used moderate-to-high-intensity exercise, five studies (24, 31, 32, 37, 39) used moderate-intensity exercise, one study (37) used low-to-moderate-intensity exercise, and two studies (28, 32) did not specify (Table 3).

**Table 3:** Exercise protocols used in the studies.

Author and Year	Type of exercise	Intensity	Volume	Exercise session	Frequency and period	Supervised or non-supervised
Balachandran 2014 (26)	Hypertrophy/strength	70% of 1-RM	110–120 min/week	The subject performed 10 repetitions. 1-RM was reached by all subjects in no more than 4–5 attempts.	2 day/w, 15 weeks	Supervised
	High-speed circuit	50–80% of 1-RM	80–90 min/week	Leg press, chest press, lat pulldown, biceps curl, leg curl, hip adduction, calf raise, shoulder press, and hip adduction.		
Kim 2016 (28)	CE	NA	180 min/week	Warm-up, weight/machine training, stationary bicycle AR, and chair/standing exercise.	3 day/w, 12 weeks	Supervised
Maltais 2016 (27)	RT	80% of 1-RM	180 min/week	Shoulder press, sit-ups, biceps curls, leg press, bench press, leg extension, rowing extensions, and leg curls (3 sets × 8 repetitions).	3 day/w, 16 weeks	NA
Vasconcelos 2016 (29)	RT	40–75% of 1-RM	120 min/week	Straight leg raises for posterior leg muscles, straight leg raise, straight leg raises with crossing.	2 day/w, 10 weeks	Supervised
Chen 2017 (31)	RT	60–70% of 1-RM	120 min/week	Shoulder presses, bicep curls, triceps curls, bench presses, deadlifts, leg swings, squats, standing rows, unilateral rows, and split front squats (3 sets × 8–12 repetitions).	2 day/w, 8 weeks	Supervised
	AR			Stepping on the spot, knee lifts, high knee running, rowing arm swings, arm swings, twist steps, arm raises, squats, V steps, mambo steps, diamond steps, and point step jumps.		
	CE			The subject performed once a week with the AR following 48 h after RT.		
Huang 2017 (32)	ERT	13 points on the RPE	165 min/week	3 sets of 10 repetitions for training the shoulders, arms, lower limbs, chest, and abdomen.	3 day/w, 12 weeks	Supervised
Liao 2017 (24)	RT	13 points on the RPE	135–150 min/week	3 sets of 10 gentle concentric and eccentric contractions are slowly performed through the full range of motion.	3 day/w, 12 weeks	Supervised
Park 2017 (33)	CE	RPE in the 13–17 range	150–240 min/week	RT included elbow flexion, wrist flexion, shoulder flexion, lateral raise, front raise, chest press, reverse flies, side bend, dead lift, squat, leg press, and ankle plantar flexion. AR involved various walking activities (sideways, backward, and forward walking, and slow and fast indoor walking).	3 day/w, 24 weeks	Supervised
Chiu 2018 (39)	RT	12–13 points of RPE	120 min/week	Upper body exercises included 3 sets of training that targeted the biceps, deltoids, grip, and pinch. Lower extremities included 3 sets of leg extensions, leg flexions, calf raises, stepping forward and sideward, and others.	2 day/w, 12 weeks	Supervised
Liao 2018 (37)	ERT	13 points on the RPE scale	165 min/week	Seated chest press, seated row, seated shoulder press, knee extension, knee flexion, hip flexion, and hip extension.	3 day/w, 12 weeks	Supervised
Nabuco 2019 (30)	RT	Increases from 2 to 5% of 1-RM for upper limb and 5 to 10% for lower limb	NA	Chest press, horizontal leg press, seated row, knee extension, preacher curl (free weights), leg curl, triceps pushdown, and seated calf raise.	3 day/w, 12 weeks	Supervised
Liao 2020 (38)	ERT	65–80% of 1-RM; 13–15 points of RPE	110 min/week	Seated chest press, seated row, seated shoulder press, knee extension, knee flexion, hip flexion, and hip extension.	2 day/w, 12 weeks	Supervised
Jung 2022 (34)	Circuit exercise	60–80% of HRR	75–225 min/week	Walking in place, shoulder press and squat, twist to dash, lunge, jumping jacks, kickback, push-up, crunch, hip bridge, and bird dog.	3 day/w, 12 weeks	Supervised
Magtouf 2023 (36)	Concurrent exercise	300 a.u.	120 min/week	Motor skill exercises (zigzag cone walking, obstacle course walk, ladder agility drills, direction change walking, obstacle relay race, zigzag ball passing, balance board zigzag walk, and cone weaving); Strengthening exercises (calf raises, toe taps, seated leg extensions, mini squats, seated leg lifts, bridge exercise, leg raises, hip abduction with resistance band); Posture exercises (single-leg balance with eyes closed, heel-to-toe walking with eyes closed, balance board or wobble board, tandem stance, toe tapping, ball toss, reaching and bending, sensory walk, picking up objects with toes).	2 day/w, 24 weeks	Supervised
Jung 2024 (35)	Circuit exercise	60–85% of HRR	135–225 min/week	Walking in place, shoulder presses and squats, twist dashes, lunges, jumping jacks, kickbacks, modified push-ups, crunches, hip bridges, and bird dogs.	3 day/w, 12 weeks	Supervised

ERT, elastic resistance training; RT, resistance training; AR, aerobic exercise; CE, combined exercise, a mix of RT and AR; 1-RM, one repetition maximum; HRmax, heart rate maximum; RPE, rating of perceived exertion; HRR, heart rate reserve; a.u., arbitrary units; NA, not available.

### 3.4 Risk of Bias in included studies

**Figure 2:**
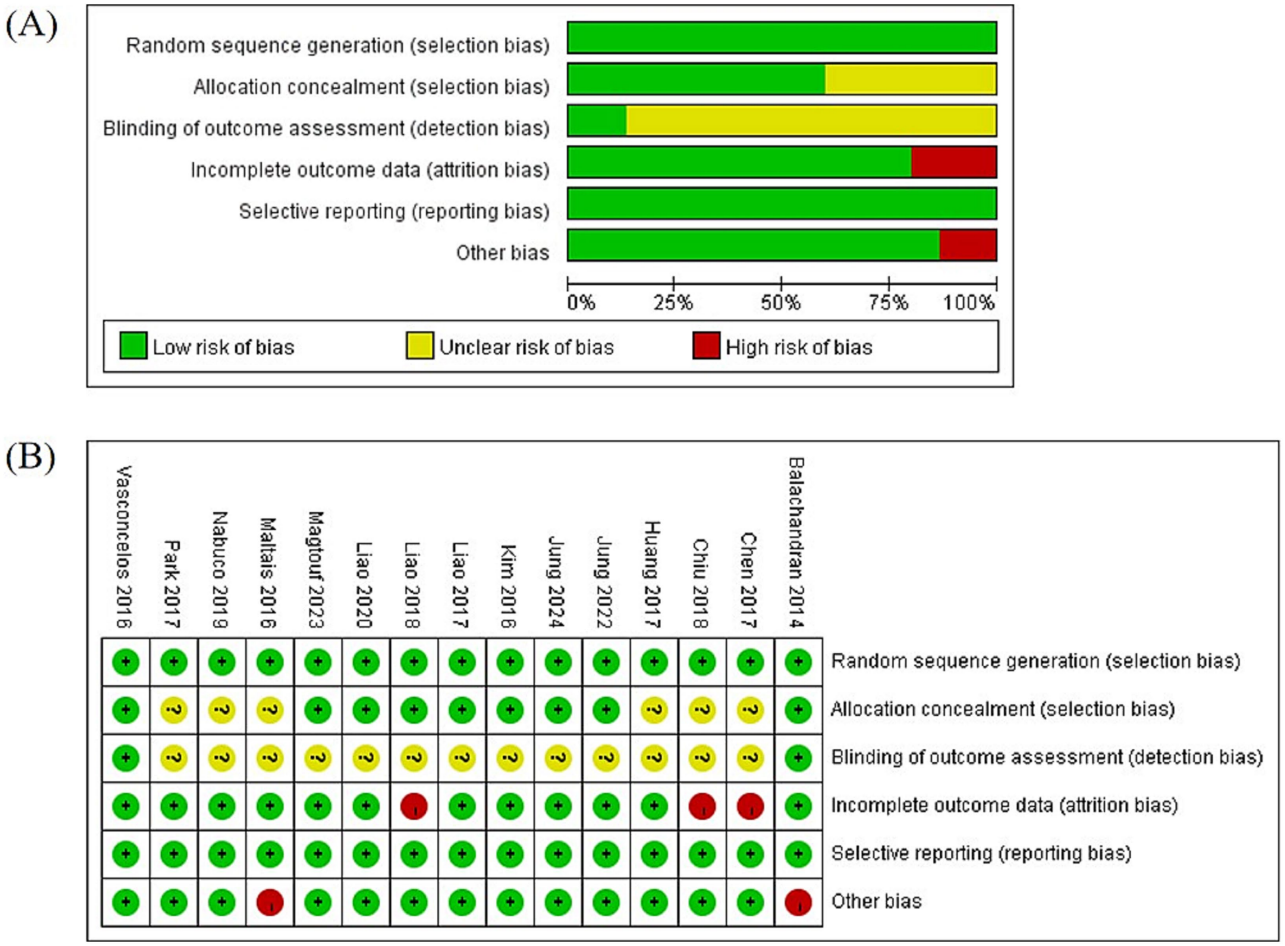
**(A)** Risk-of-bias graph. **(B)** Risk-of-bias summary.

The quality assessment was performed according to the Cochrane Bias Risk Assessment Tool. Nine studies (24, 26, 28, 29, 34–38) described allocation concealment, whereas others had unclear allocation concealment. Two studies (22, 26) used blinding of assessors, whereas 13 did not. All studies reported attrition rates. A data attrition rate exceeding 20% was regarded as high risk, except when an intention-to-treat analysis was performed. Three studies (31, 33, 39) were considered high risk, whereas the others were considered low risk. Studies were evaluated as high risk if they had a small sample size (fewer than 10 participants in any group), lacked supervision, or had large measurement errors in outcome assessment. One study (26) was assessed as high risk due to its small sample size, and another study was at high risk due to unclear supervision and small sample size. Blinding of participants and personnel was not evaluated because of the nature of the exercise interventions. Overall, 15 studies had a relatively low risk of bias (Figure 2).

### 3.5 Effects of exercise on body composition

#### 3.5.1 Effects of exercise on BMI and BF%

**Figure 3:**
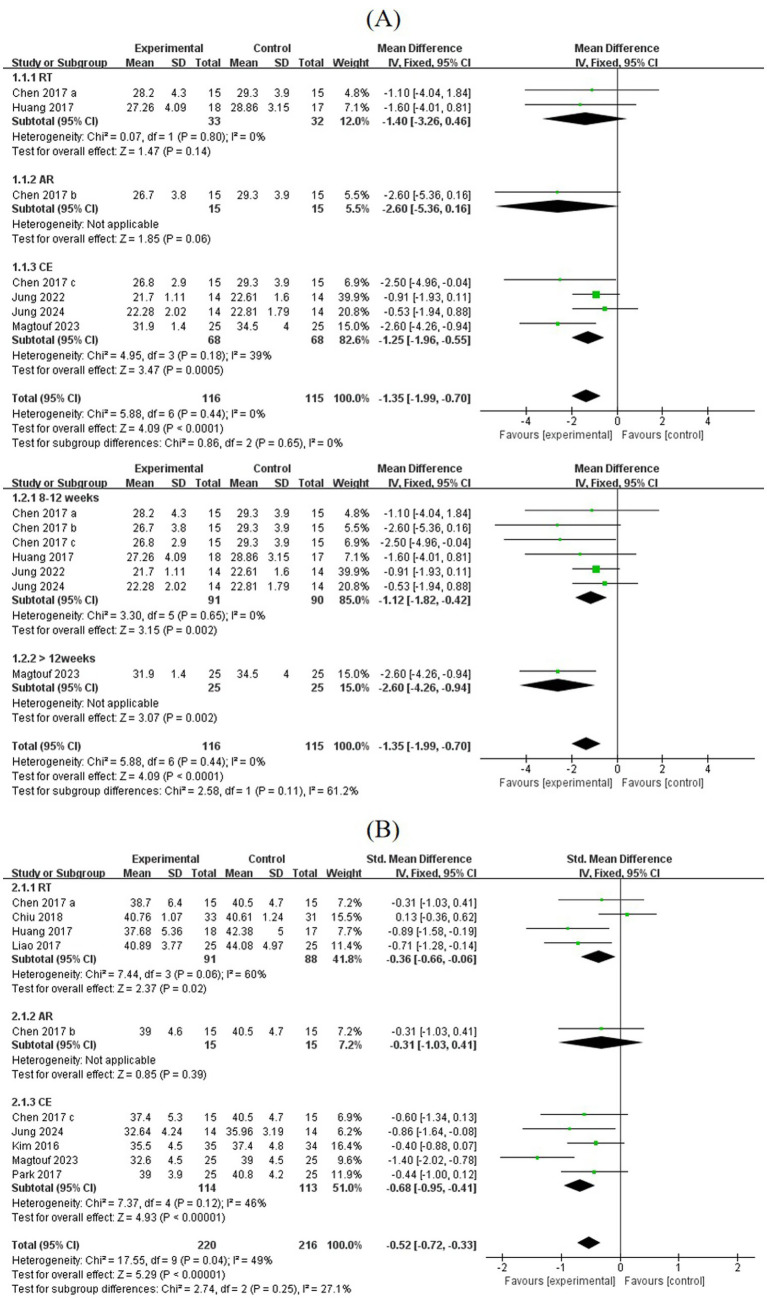
**(A,B)** Forest plots of comparisons between exercise and the control groups on BMI and BF%.

Based on five studies, exercise significantly reduced BMI (MD = −1.35, 95% CI: [−1.99, −0.70], *p* < 0.0001). Subgroup analysis showed that CE significantly reduced BMI (MD = −1.25, 95% CI: [−1.96, −0.55], *p* < 0.001), AR showed a near-significant reduction (MD = −2.60, 95% CI: [−5.36, 0.16], *p* = 0.06), and RT did not reduce BMI (MD = −1.40, 95% CI: [−3.26, 0.46], *p* = 0.14). Exercise lasting 8–12 weeks (MD = −1.12, 95% CI: [−1.82, −0.42], *p* = 0.002) and >12 weeks (MD = −2.60, 95% CI: [−4.26, −0.94], *p* = 0.002) both significantly reduced BMI. BF% was decreased (MD = −0.52, 95% CI: [−0.72, −0.33], *p* < 0.00001). Both CE and RT significantly reduced BF% (CE: MD = −0.68, 95% CI: [−0.95, −0.41], *p* < 0.00001; RT: MD = −0.36, 95% CI: [−0.66, −0.06], *p* < 0.05), while AR showed no significant difference (MD = −0.31, 95% CI: [−1.03, 0.41], *p* = 0.39) (Figure 3).

#### 3.5.2 Effects of exercise on fat mass, ASM, and ASMI

**Figure 4:**
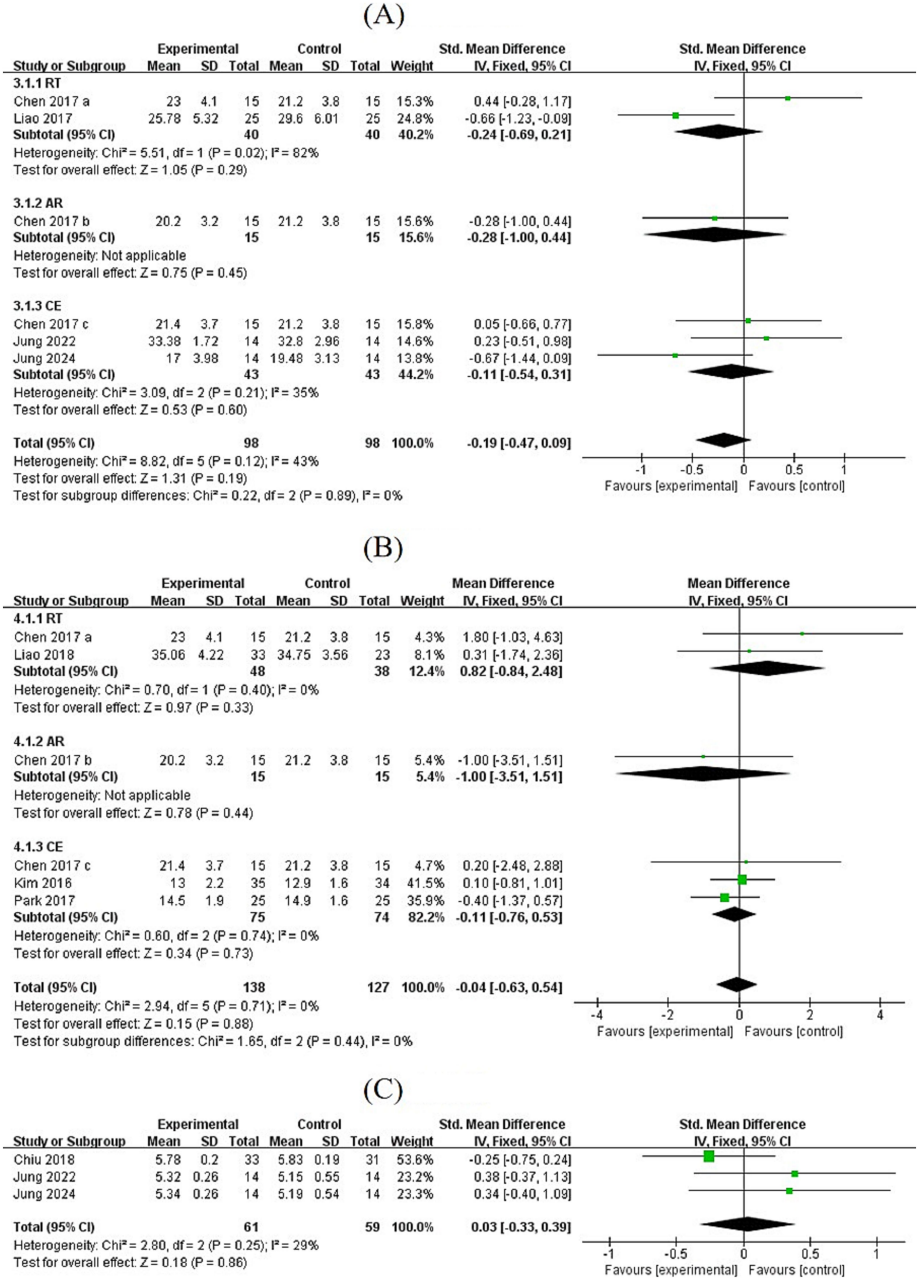
**(A)** Forest plots of comparisons between exercise and control groups in fat mass; **(B,C)** forest plots of comparisons between exercise and control groups in ASM and ASMI.

Four studies reported data on fat mass, involving 98 SO patients. The pooled effect size showed that the overall fat mass was not significantly reduced after exercise (MD = −0.19, 95% CI: [−0.47, 0.09], *p* = 0.19). A total of four studies reported appendicular skeletal muscle mass (ASM), involving 138 participants. A total of three studies reported appendicular skeletal muscle mass index (ASMI), involving 61 SO patients. No significant differences were found in ASM (MD = −0.04, 95% CI: [−0.63, 0.54], *p* = 0.88) and ASMI (MD = 0.03, 95% CI: [−0.33, 0.39], *p* = 0.86), suggesting that muscle mass remained largely unchanged following the intervention (Figure 4).

### 3.6 Effects of exercise on physical function

#### 3.6.1 Effects of exercise on grip strength and gait speed

**Figure 5:**
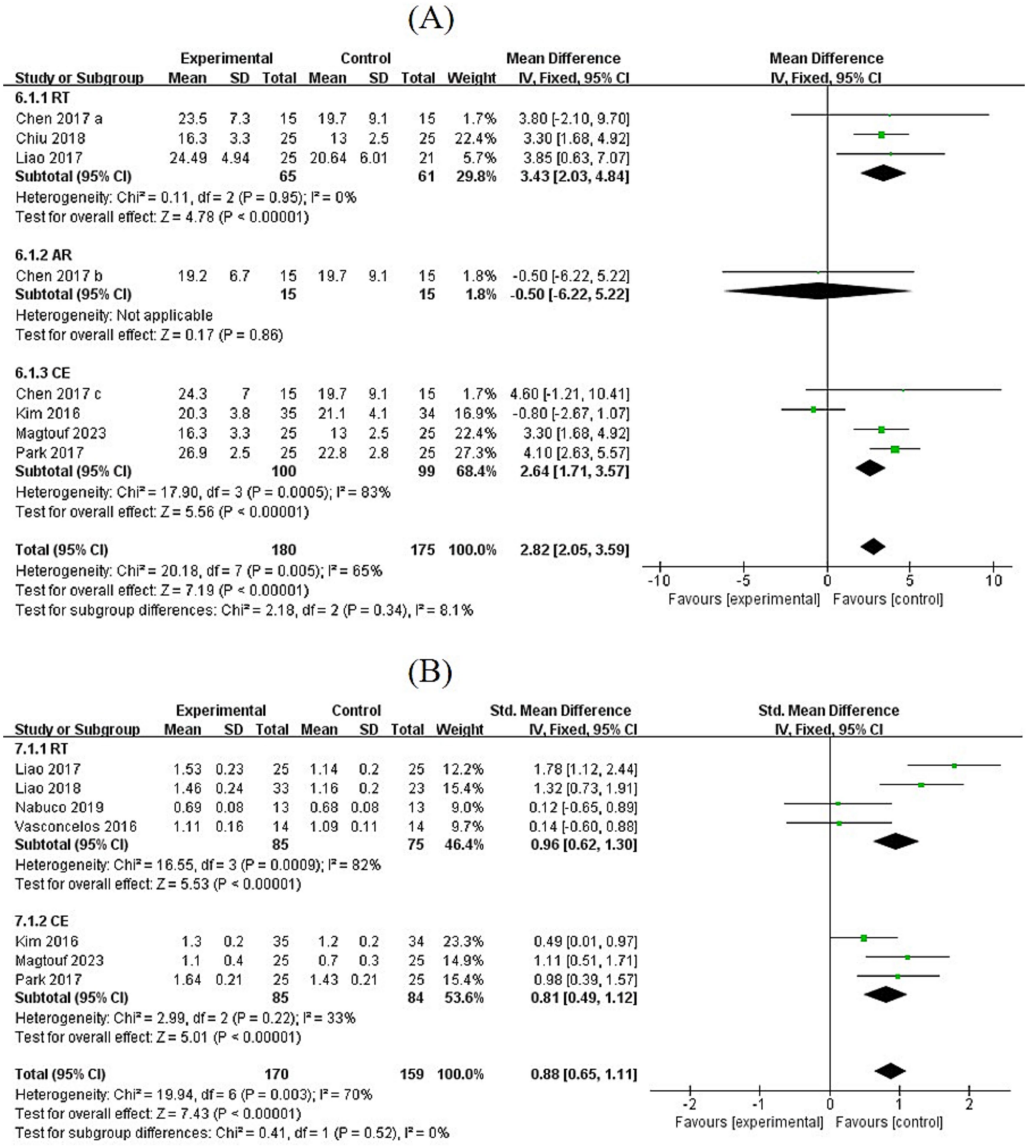
**(A,B)** Forest plots of comparisons between exercise and the control groups in grip strength and gait speed.

Based on the pooled analysis of six studies, grip strength increased after the intervention (MD = 2.82, 95% CI: [2.05, 3.59], *p* < 0.00001). The effects of the different exercise modalities varied. Both RT and CE significantly increased grip strength (RT: MD = 3.43, 95% CI: [2.03, 4.84], *p* < 0.00001; CE: MD = 2.64, 95% CI: [1.71, 3.57], *p* < 0.00001), while AR did not (MD = −0.50, 95% CI: [−6.22, 5.22], *p* = 0.86). Seven studies involving 170 participants evaluated 6-min gait speed and habitual walking speed. The results showed that gait speed improved after exercise intervention (MD = 0.88, 95% CI: [0.65, 1.11], *p* < 0.00001). Both RT and CE significantly increased gait speed (RT: MD = 0.96, 95% CI: [0.62, 1.30], *p* < 0.00001; CE: MD = 0.81, 95% CI: [0.49, 1.12], *p* < 0.00001) (Figure 5).

#### 3.6.2 Effects of exercise on TUG and knee extension

**Figure 6:**
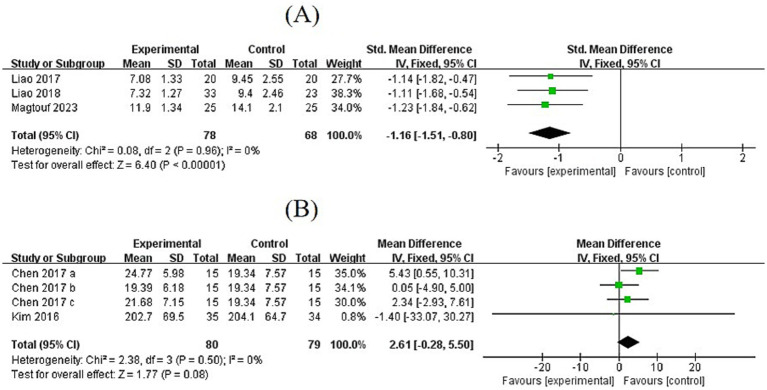
**(A,B)** Forest plots of comparisons between exercise and the control groups in TUG and knee extension.

Three studies evaluated the timed Up and Go (TUG) test. The pooled effect size showed that TUG time was significantly shortened after exercise in SO patients (MD = −1.16, 95% CI: [−1.51, −0.80], *p* < 0.00001). Exercise showed a trend toward improving knee extension strength (MD = 2.61, 95% CI: [−0.28, 5.50], *p* = 0.08), indicating potential improvements in daily activity function and exercise endurance (Figure 6).

### 3.7 Effects of exercise on glucose and lipid metabolism

#### 3.7.1 Effects of exercise on glucose and insulin

**Figure 7:**
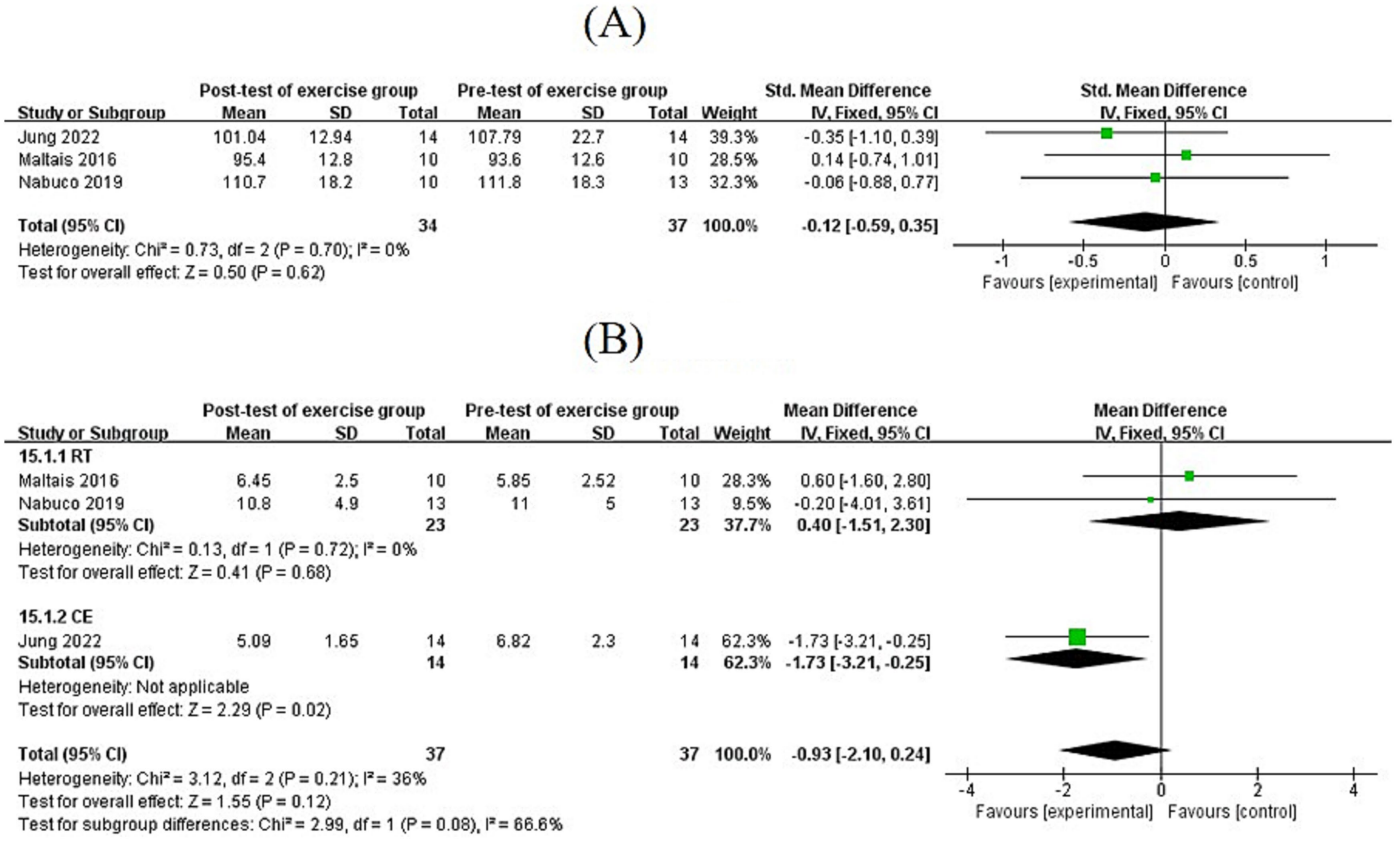
**(A,B)** Forest plots of post-test and pre-test of exercise group in glucose and insulin.

Among the included studies, researchers mainly focused on within-group differences before and after exercise, and most did not assess between-group differences after the intervention. Therefore, we analyzed within-group differences before and after the exercise. It was found that glucose and insulin levels decreased after the exercise intervention in SO patients, but not significantly (glucose: MD = −0.12, 95% CI: [−0.59, 0.35], *p* = 0.62; insulin: MD = −0.93, 95% CI: [−2.10, 0.24], *p* = 0.12). Interestingly, CE significantly reduced insulin levels in SO subjects (MD = −1.73, 95% CI: [−3.21, −0.25], *p* < 0.05) (Figure 7).

#### 3.7.2 Effects of exercise on TC and TG, HDL-C, and LDL-C

The pre- to post-test differences in TC in the exercise group confirmed a significant reduction (MD = −0.38, 95% CI: [−0.71, −0.06], *p* = 0.02). Pooled results from three studies showed that exercise intervention had a positive but non-significant effect on TG (MD = −1.58, 95% CI: [−7.22, 4.06], *p* = 0.58).

**Figure 8:**
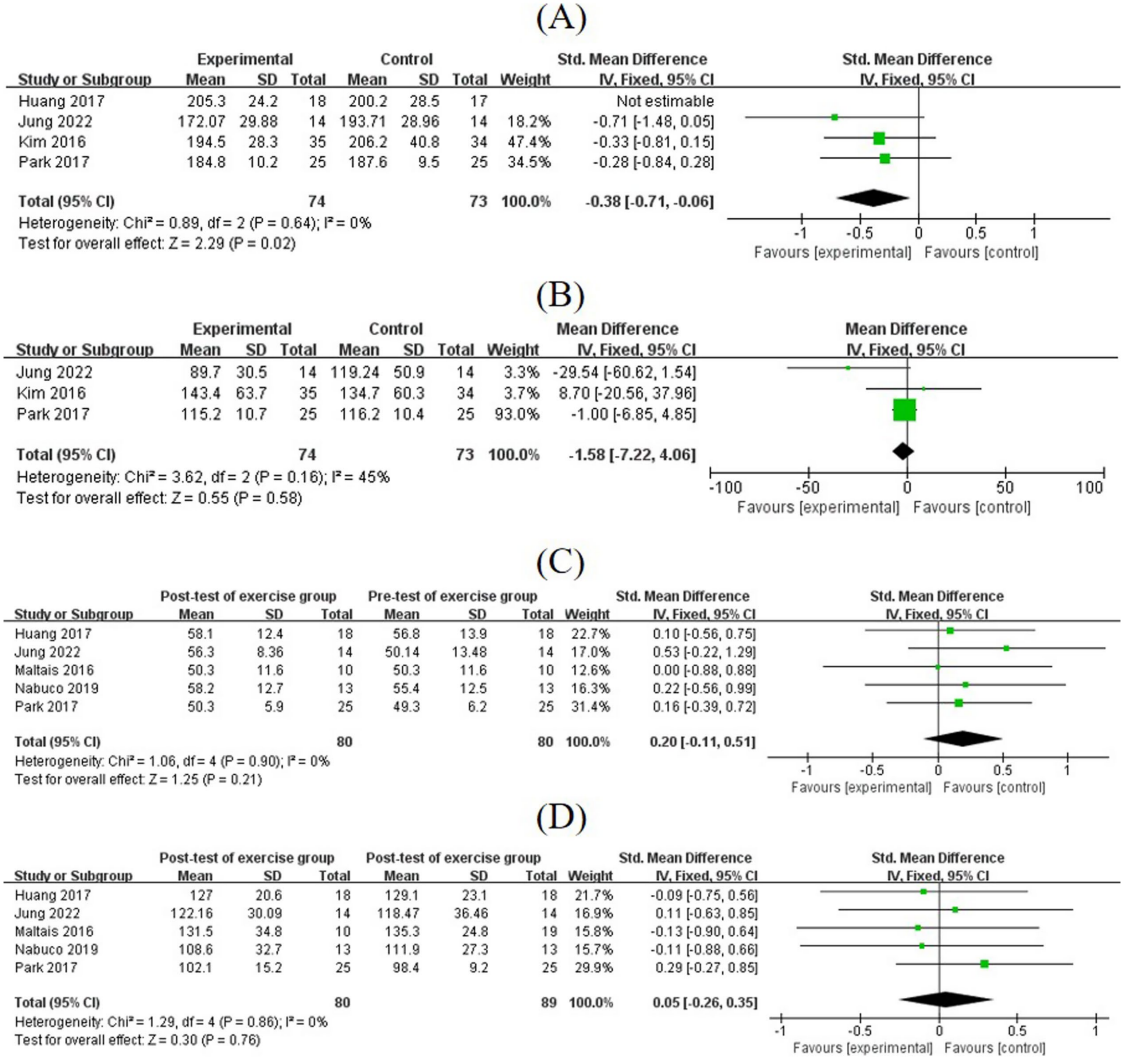
**(A)** Forest plots of comparisons between the post-test and pre-test of the exercise group in the TC. **(B)** Forest plots of comparisons between exercise and control groups in the TG. **(C,D)** Forest plots of post- and pre-test comparisons of HDL-C and LDL-C levels in the exercise group.

Pooled results from three studies showed a positive but non-significant effect of exercise on HDL-C (MD = 0.20, 95% CI: [−0.11, 0.51], *p* = 0.21). Pooled results from five studies showed a positive but non-significant effect of exercise on LDL-C (MD = 0.05, 95% CI: [−0.26, 0.35], *p* = 0.76) (Figure 8).

### 3.8 Effects of exercise on inflammatory biomarkers

**Figure 9:**
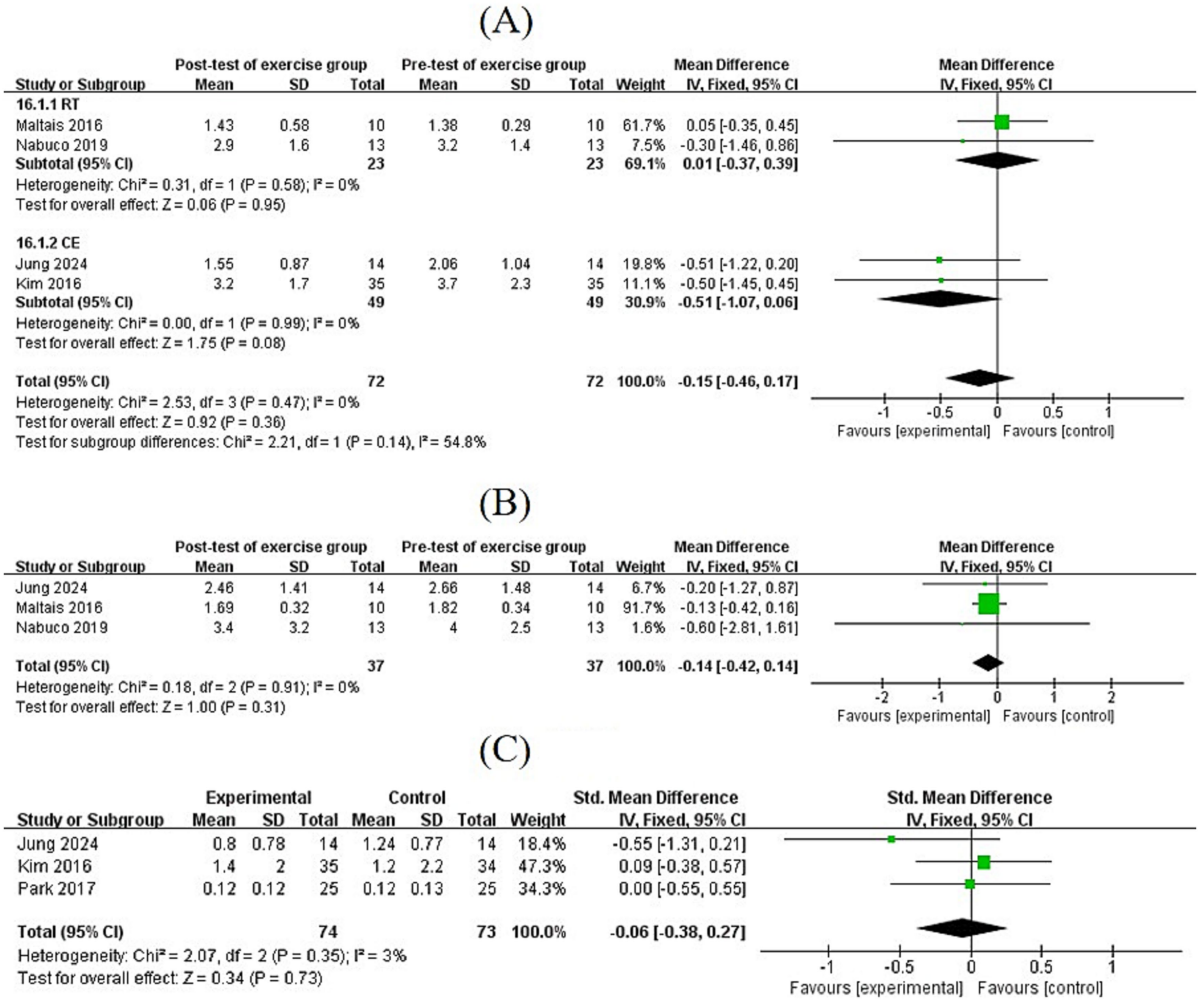
**(A,B)** Forest plots of post-test and pre-test of IL-6 and TNF-*α* levels in exercise groups. **(C)** Forest plots of comparisons of CRP levels in the exercise and control groups.

Five studies assessed inflammatory biomarkers, including IL-6, TNF-*α*, and CRP levels. The pooled effect size for IL-6 was MD = −0.15 (95% CI: [−0.46, 0.17], *p* = 0.36), for TNF-*α* was MD = −0.14 (95% CI: [−0.42, 0.14], *p* = 0.31), and for CRP was MD = −0.06 (95% CI: [−0.38, 0.27], *p* = 0.73). Subgroup analysis of IL-6 levels showed that CE tended to reduce IL-6 levels, although not significantly (MD = −0.51, 95% CI: [−1.07, 0.06], *p* = 0.08), a finding that may reach significance in larger cohorts (Figure 9).

## 4 Discussion

Both obesity and sarcopenia are common chronic conditions. When they occur concurrently, the condition known as SO emerges, with a synergistic effect that can lead to severe consequences (2). Currently, although there have been relatively thorough investigations into SO populations, many issues remain to be explored in depth. Exercise has emerged as a pivotal intervention in the management of SO; however, challenges persist in optimizing exercise interventions for SO (23). The optimal exercise intensity, frequency, and duration remain to be precisely defined. Different studies have adopted varying protocols, making it difficult to establish a standardized exercise prescription. Additionally, the heterogeneity among SO patients, considering factors such as age, sex, baseline physical condition, and comorbidities, further complicates this issue. For instance, studies have begun to explore the benefits of high-intensity interval training for comorbidities in older adults (40). Early intervention appears to be a critical treatment window for older SO patients, and the specific benefits of different exercise types and durations require further research.

### 4.1 Effects of exercise on body composition in stage *I* SO

Excessive fat accumulation, especially abdominal fat, can lead to insulin resistance, increasing the risk of obesity and chronic diseases such as type 2 diabetes and cardiovascular diseases (41). Therefore, reducing fat mass is critical for alleviating the metabolic burden in patients with SO. The significant reduction in BMI following exercise intervention (MD = −1.35, *p* < 0.0001) confirms the negative energy balance theory, whereas subgroup analyses highlight clinically relevant differences in exercise modality effects. CE synergizes aerobic metabolism and muscle synthesis, enhancing BMI reduction compared to RT or AR alone. Both 8–12 weeks and >12 weeks of exercise significantly reduced BMI, with a difference in effect sizes (MD = −1.12 vs. MD = −2.60), suggesting a potential intervention duration threshold. Short-term exercise is characterized by rapid fat loss, whereas long-term interventions optimize body composition through muscle redistribution (42). From a clinical perspective, even modest reductions in BMI are directly associated with alleviated obesity-related health risks. Specifically, when BMI decreases from the obese range (≥30 kg/m^2^) to the overweight range (25–29.9 kg/m^2^), accompanied by improvements in metabolic parameters such as blood pressure, fasting glucose, and lipid profiles, it indicates a substantial reduction in the patient’s susceptibility to complications including cardiovascular diseases and type 2 diabetes. Furthermore, such reductions in BMI contribute to decreased joint loading (thereby mitigating symptoms of osteoarthritis) and amelioration of obesity-associated conditions such as sleep apnea. These changes are of particular clinical relevance in elderly patients with stage *I* SO: they not only reduce the risk of disease progression but also enhance activities of daily living and quality of life, thus representing meaningful clinical outcomes rather than merely numerical changes.

Notably, the pooled effect on fat mass was non-significant (MD = −0.19, *p* = 0.19), and changes in ASMI and ASM were also not significant (*p* = 0.86 and *p* = 0.88, respectively), suggesting that exercise has a greater impact on fat distribution than on muscle mass. Notably, skeletal muscle mass remained unchanged, suggesting that exercise intensity or inadequate protein intake limits muscle synthesis. The significant decrease in BF% (MD = −0.52) contrasts with the non-significant change in fat mass (MD = −0.19), which may stem from (i) BF% calculations relying on whole-body composition, while fat mass analyses are limited by differences in local fat distribution, and (ii) the absence of a concomitant increase in muscle mass, leading to a decrease in BF% without a significant change in absolute fat mass. RT reduces BF% by 0.36 (MD = −0.36), corroborating that RT indirectly promotes lipolysis by increasing basal metabolic rate through lean body mass. This effect was weaker than that of CE (MD = −0.68), indicating that the aerobic component makes a more prominent immediate contribution to fat oxidation. Further research is needed to explore factors interfering with the RT-BMI link, such as how compensatory muscle growth influences weight measurements.

Muscle mass typically decreases gradually with age, starting at approximately 30 years and accelerating after 60 years (43). Among the three exercise modalities, RT, the most targeted for stimulating muscle growth, only shows an increasing effect on muscle mass, yet it has not reached statistical significance. This could potentially be attributed to multiple factors. For instance, if training intensity does not reach the threshold required to stimulate muscle growth, or if exercise selection does not comprehensively target muscle groups, effective muscle growth is unlikely. RT intensities in the studies ranged from 40 to 80% 1-RM; however, the combined effect showed no significant effect of RT on ASM (MD = 0.82, *p* = 0.33), suggesting that most studies may have used low-to-moderate intensities (<60% 1-RM), which are insufficient to activate key signaling pathways for muscle protein synthesis, such as mTOR. For muscle growth, resistance training requires ≥70% 1-RM to significantly stimulate satellite cell activation (44), and some protocols in the included studies may not have exceeded this threshold. CE was effective in reducing BMI (MD = −0.67) but had no significant effect on muscle mass, possibly due to insufficient volume of resistance training because AR takes up too much of the training time or is performed less frequently. In addition, growth hormones such as testosterone are crucial for muscle growth, and individual differences in hormone levels may lead to varying effects (45). Future research should focus on optimizing RT protocols based on individual patient differences (e.g., age, health status, and hormone levels) and precisely formulating training intensity, frequency, and exercise combinations.

### 4.2 Effects of exercise on physical function in stage *I* SO

Physical activity ability is directly linked to enhanced independence in SO patients, as it reduces dependence on assistive devices and expands social activity scope. Regarding exercise-induced improvements in muscle strength, endurance, and physical function in SO patients, both RT and CE significantly enhance grip strength and gait speed, whereas AR shows no similar effect. Grip strength, a core indicator for sarcopenia diagnosis, improves significantly after RT (MD = 3.43) and CE (MD = 2.64) interventions, confirming that resistance training is the gold standard for improving muscle strength. Mechanistically, RT stimulates muscle fiber recruitment and satellite cell activation through mechanical loading, whereas CE indirectly enhances muscular endurance by improving oxygen supply and energy metabolism (46). This can be attributed to RT triggering muscle protein synthesis via resistance contraction, whereas CE improves cardiopulmonary function, enhances muscle oxygenation, and promotes strength development.

AR shows no significant effect on grip strength (MD = −0.50, *p* = 0.86), indicating that aerobic metabolism alone is ineffective in stimulating muscle protein synthesis. This aligns with the histological findings indicating no alteration in the ratio of skeletal muscle fast-twitch fibers. In physical function tests, seven studies demonstrate that exercise interventions improve gait speed by 0.88 (*p* < 0.00001), and three studies on the TUG test show that exercise shortens TUG time by 1.16 s (*p* < 0.00001), reflecting improved daily activity ability. Knee extension strength shows a marginal improvement (MD = 2.61, *p* = 0.08), potentially limited by (i) insufficient sample size (only three studies) and (ii) variations in testing methods (isometric vs. isotonic contraction). This suggests that larger sample sizes are needed to validate exercise effects on endurance and functional activities. An in-depth analysis of knee joint characteristics and muscle activation sequences during exercise can optimize specific details (e.g., adjusting flexion-extension angles and force timing in knee extension exercises) to develop modules for improving knee function.

Notably, although ASMI shows no significant change, the dissociation between functional improvement and muscle mass suggests that exercise enhances function through neuromuscular coordination rather than pure muscle fiber hypertrophy. This finding provides a theoretical basis for exercise prescriptions in frail elderly populations. Future research should explore integrated exercise programs, for example, low-intensity AR to improve endurance and warm up joints, followed by gradual RT to build strength, or CE with a longer RT duration than AR to enhance overall activity and self-care ability.

### 4.3 Effects of exercise on metabolic and inflammatory regulation mechanisms in stage *I* SO

Skeletal muscle is the primary organ responsible for handling postprandial glucose via insulin-dependent mechanisms, thus playing a crucial role in regulating glucose homeostasis (47). After food intake, as blood glucose increases, insulin binds to its receptor, activating receptor tyrosine kinase and triggering intracellular signal transduction (48). When insulin resistance occurs, it reflects that insulin signal transduction is impaired, leading to reduced glucose utilization in muscle tissue and affecting muscle protein synthesis. Therefore, impaired glucose transport and metabolism are closely related to SO. In the long-term state of IR, the translocation of GLUT4 to the cell membrane is blocked, resulting in decreased glucose uptake by the muscles (49). Exercise can promote the expression and translocation of GLUT4 in skeletal muscle cells, enabling more effective glucose entry into cells for utilization and increasing glucose uptake to maintain blood glucose balance (50). Metabolic outcomes show that while exercise-induced glucose reduction is non-significant (MD = −0.12, *p* = 0.62), CE significantly lowers insulin levels (MD = −1.73, *p* < 0.05), highlighting its prominent role in improving insulin sensitivity. Mechanistically, the aerobic component activates the AMPK pathway to promote GLUT4 translocation to the cell membrane, whereas resistance training enhances insulin receptor sensitivity through mechanical stress, thereby forming a metabolic regulatory cascade (51). In contrast, the non-significant glucose reduction (*p* = 0.62) may relate to baseline glucose level variations and compensatory hepatic gluconeogenesis following exercise.

The effect of exercise on blood lipids in SO is mainly manifested in the reduction of TC. Lipid regulation showed a TC priority pattern, with a decrease in TC by 0.38 mg/dL (*p* < 0.05), but no statistically significant changes in TG, HDL-C, and LDL-C levels (*p* = 0.58, 0.21, and 0.76, respectively), suggesting that the regulation of the lipid profile by exercise is selective. Mechanistically, CE may promote glucose uptake through activation of the AMPK pathway, and its regulatory effect on insulin is consistent with previous studies showing increased expression of the GLUT4 transporter protein in skeletal muscle. It may also be associated with the exercise-induced selective elevation of lipoprotein lipase (LPL) activity. LPL preferentially hydrolyzes TC-rich lipoproteins, whereas TG metabolism depends on hepatic lipase, which remains underactivated during moderate-intensity exercise (52). This finding explains why TC is more sensitive to exercise and suggests that high-intensity interval training may be more effective in improving TG levels, which needs to be verified in future studies. For patients with glucose metabolism disorders, CE is recommended along with precise blood glucose monitoring and personalized exercise duration or frequency combinations for glycemic control. An intervention of low-, moderate-, and high-intensity AR carried out among overweight and dyslipidemic individuals indicated that exercise was beneficial for various lipid and lipoprotein variables, and this was most evident with high-intensity exercise (53). Kraus et al. (53) also pointed out that these improvement effects were mainly related to the amount of activity, rather than the intensity of exercise or the improvement of health conditions. Hence, in-depth research on the correlation between exercise intensity, duration, and dynamic changes in blood lipid indicators should be carried out to refine exercise lipid-lowering strategies, providing a scientific basis for the SO population to choose the exercise duration.

In the aging process, sarcopenia stems not only from mild chronic inflammation but also from fiber-intrinsic defects (54). Disorders in blood glucose and blood lipids are correlated with the inflammatory response, which is enhanced in the state of chronic hyperglycemia (55). An increasing amount of evidence shows that the mutual inflammation between adipose tissue and skeletal muscle is a major contributing factor to SO (56). The fat accumulation in muscle tissue promotes an inflammatory cascade and oxidative stress, leading to mitochondrial dysfunction, impaired insulin signaling, and muscle atrophy; the interaction between myokines and adipokines results in a negative feedback loop to further exacerbate SO and insulin resistance (18). The included studies showed that exercise caused the inflammatory biomarkers, including IL-6, TNF-*α*, and CRP, in the SO population to decrease, but without significant differences. The lack of significant changes in inflammatory factors aligns with the clinically observed “metabolic-inflammatory decoupling” phenomenon, whereby exercise first improves metabolic markers and subsequently suppresses inflammation indirectly through metabolites such as adiponectin (57). The reduction in IL-6 by CE may achieve statistical significance in populations with high baseline IL-6 levels, reflecting a threshold-dependent anti-inflammatory response, where exercise-mediated inflammation suppression becomes apparent only when baseline inflammation exceeds a critical threshold (58). Thus, continuous monitoring of inflammatory factors serves as a barometer for exercise efficacy to adjust intervention cycles.

### 4.4 Methodological limitations and heterogeneity considerations

Nevertheless, this meta-analysis had certain limitations. Although subgroup analyses were performed, it might still not be possible to fully account for all sources of heterogeneity. Due to the limitations in the number and quality of the studies, it was difficult to draw a definite conclusion regarding the exercise intervention effect on stage *II* SO patients, and only stage *I* SO patients were taken as the main research subjects. During the data analysis process, because of the different focuses of the included studies, the forest plots of some metabolic indicators and inflammatory markers mainly showed within-group comparisons before and after the exercise intervention, and the between-group differences between the intervention and control groups after exercise were not presented. This is also a key point that needs to be further considered in subsequent studies.

This study has three primary limitations: (i) Most RCTs concentrate on between-group differences before and after intervention, yet some studies only focus on within-group pre-post differences, which renders the existing evidence only able to prove that exercise is superior to baseline, while making it difficult to precisely define the relative advantages of different exercise modalities. (ii) The small sample size for certain indicators (involving merely 3–4 studies) might introduce bias in effect size estimation. For example, the pooled effect of 98 patients in the fat mass analysis approached significance (*p* = 0.19), suggesting that an insufficient sample size could mask the true effect. (iii) The high heterogeneity in exercise protocols (such as RT intensity ranging from 40 to 80% 1-RM and two to five sessions per week) compromises the external validity of the results, restricting the universality of clinical exercise prescriptions. (iv) The unfocused nature of the role of nutrition is a limitation; therefore, we may analyze the benefits of the combined approach to exercise and nutrition interventions.

The heterogeneity test showed that the I^2^ was 66.6% for insulin analysis and 54.8% for IL-6 analysis. The funnel plot analysis of these two indicators was attributed to the inclusion of Maltais’ studies (24), primarily stemming from (i) the insufficient number of included studies, with only three studies (*n* = 37) in the insulin analysis and four studies (*n* = 72) in the IL-6 analysis, which might have introduced bias in the effect size estimation. (ii) Variations in the definition of obesity and bias in the obese populations included across studies, leading to heterogeneous degrees of muscle loss among SO populations in different studies. (iii) Most included studies adopted moderate-intensity exercise, whereas high-intensity resistance training could promote anabolism through stronger muscle damage responses, while low-intensity regimens showed limited effects. The non-significant results for decreased metrics might have overrepresented negative studies, with positive findings potentially missed because of non-publication bias.

## 5 Future research directions

The clinical definition of SO has not been unified, which limits its wide application in clinical practice and large-scale epidemiological research. With the continuous deepening of research on SO, an increasing number of researchers no longer define obesity solely based on BMI but gradually use BF% instead, indicating that the precision of research is constantly improving. Currently, there are various methods for evaluating muscle mass and strength according to the EWGSOP2 for defining sarcopenia (17). Decisive indicators include a reduction in muscle mass and muscle strength, regardless of the magnitude and degree of obesity. Therefore, preventive and treatment measures mainly rely on improving insulin resistance and dyslipidemia caused by increased fat mass, as well as the decline in muscle function and the risk of injury due to decreased muscle mass. Due to the natural reduction in muscle mass and strength and age-related changes in fat distribution, the diagnostic criteria should vary among different age groups. For the elderly, especially the very old, because of the more obvious changes in body composition and physiological functions, the diagnostic threshold should be appropriately loosened to improve the accuracy and sensitivity of the diagnosis. In the future, more standardized, easy-to-operate, and cost-effective diagnostic tools should be developed to identify patients with SO at an early stage.

Given the limitations of this study, future research should construct a biomarker-exercise type-efficacy prediction model: (i) Stratify patients based on baseline IL-6 levels, recommending CE (≥3 sessions/week, 45 min/session) for those with high inflammatory load and RT (2-3 sessions/week, 60–80% 1-RM) for low-inflammatory individuals. (ii) Explore exercise-nutrition interventions, such as RT combined with leucine supplementation. Leucine, as an mTOR pathway activator, may offset the limitations of single RT in promoting muscle mass gain. (iii) Conduct multicenter, large-sample RCTs (target *n* ≥ 500) with ≥24-week intervention, using a four-arm design (CE, RT, AR, and control) while monitoring muscle biopsies (PGC-1α, AMPK) and inflammatory factor dynamics.

Overall, exercise interventions for stage *I* SO involve complex issues with multiple factors. In-depth studies on its pathological manifestations and the establishment of effective exercise interventions are important for improving the health of the elderly and reducing the risk of chronic diseases. When designing future studies, confounding factors should be strictly controlled, such as basic nutritional status and whether concurrent medications affect muscle metabolism. For the elderly stage *I* SO population, early exercise intervention may play a better role, and the choice of exercise intensity should be evaluated according to the patients’ conditions. Future research should focus on multidisciplinary cooperation and integrating basic research and clinical practice to provide a stronger basis for the prevention and treatment of SO.

## 6 Conclusion

≥8-week exercise improves body composition in stage *I* SO, with CE being the most effective for fat loss. Physical function improves with both RT and CE, and RT is better for muscle strength, while CE benefits metabolism and inflammation. We recommend that CE (≥3 times/week, 45 min/session) be used for high inflammation and RT (2–3 times/week, 60–80% of 1-RM) for low inflammation. Based on observed data trends, promoting a CE model of three aerobic exercises + two RT sessions weekly is advisable, with the intensity adjusted to 40–50% 1-RM for stage *I* elderly patients. Future research needs large-sample, long-term RCTs with subgroup analyses and exercise-nutrition combinations.

## Data Availability

The original contributions presented in the study are included in the article/supplementary material, further inquiries can be directed to the corresponding author.
